# Design and evaluation of anisotropic octet-truss scaffolds for mimicking the mechanical behavior of human jawbone

**DOI:** 10.1038/s41598-025-27136-0

**Published:** 2025-11-07

**Authors:** Mahdi Kazemi, Sepehr Sheikh mahboobi, Sepideh Maralbashi

**Affiliations:** 1 Department of Mechanical Engineering, Isfahan University of Technology, Isfahan, 8415683111 Iran; 2 Department of Immunology, Faculty of Medicine, Tabriz University of Medical Science, Tabriz, Iran

**Keywords:** 3D bioprinting, Anisotropic scaffold, Trabecular bone, Young’s modulus, Immunomodulation, Macrophage polarization, Biotechnology, Engineering, Materials science, Medical research

## Abstract

The human jawbone exhibits anisotropic mechanical behavior due to its complex trabecular microstructure, creating challenges for bioinspired scaffold design in maxillofacial implants. In this study, anisotropic scaffolds were fabricated from PolyLactic Acid (PLA) using Fused Deposition Modeling (FDM) and based on modified octet-truss unit cells. Geometric anisotropy was introduced by stretching the unit cell in one direction, generating configurations with varied cell sizes and stretch ratios while maintaining constant scaffold volume. Quasi-static compression tests characterized Young’s modulus and yield strength in longitudinal and transverse orientations. Directional elongation significantly influenced anisotropy, with structures of larger unit cell size and moderate stretch ratio (e.g., 1.5 stretch with 9 mm cell size) closely approximating mandibular trabecular bone. These scaffolds achieved anisotropy ratios exceeding 2.5 between principal directions, while density values remained within the physiological jawbone range. Beyond mechanics, the immune response is equally decisive. Although PLA is widely used for its printability and biocompatibility, its degradation may acidify the microenvironment and favor pro-inflammatory macrophage activation. Strategies such as incorporating hydroxyapatite, bioactive coatings, or chemical modifiers can promote M2 polarization, enhancing angiogenesis and bone repair. Thus, the proposed scaffolds unite mechanical fidelity with immune-instructive potential for mandibular regeneration.

## Introduction

Bone tissue—particularly trabecular bone in the maxillofacial region—exhibits complex anisotropic mechanical behavior, a result of its heterogeneous microstructure and highly oriented porous network^1,2^. This directional behavior is vital for distributing stresses and for the bone’s ability to adapt to external mechanical loads^3^. For this reason, scaffolds intended for bone regeneration or replacement, especially in applications such as dental implants and maxillofacial reconstruction, must not only match the stiffness of native bone but also reproduce its anisotropic mechanical characteristics^4^.

Over the last decade, Additive Manufacturing (AM) techniques, particularly Fused Deposition Modeling (FDM), have revolutionized scaffold fabrication by enabling precise control over geometry, porosity, and material deposition^5^. This progress has opened the way for engineering anisotropic scaffolds tailored to specific biomechanical demands^6^. Among various lattice architectures, the octet-truss lattice has drawn particular attention due to its stretch-dominated deformation behavior, high specific strength, and tunable anisotropy via unit-cell modification^7^.

Different strategies have been proposed to introduce anisotropy into bone scaffolds. (1) Computational and inverse-design methods: Liu et al.^8^, for example, presented a machine-learning-based inverse design framework that can generate scaffolds with target anisotropic properties. While flexible, such approaches require advanced optimization tools and substantial computational resources. (2) Porosity-graded and density-graded scaffolds: Cheikho et al.^9^ introduced scaffolds with graded porosity to adjust anisotropy, while Zhu et al.^10^ produced cryo-printed scaffolds with anisotropic features that enhanced osteogenic activity. These methods are effective but rely on specialized techniques, such as cryo-printing, that are not widely available. (3) Bioinspired hierarchical and micro patterned scaffolds: designs inspired by natural wood^11^ or micro patterned biomimetic structures^12^ have shown that anisotropy can encourage bone ingrowth and improve biological outcomes. Similarly, anisotropic hydrogels^13^ have been developed to provide directional cues for tissue regeneration. (4) Compensation strategies for weaker architectures: for example, gyroid scaffolds have been reinforced in certain directions to overcome their inherent weaknesses^14^. Although promising, such strategies often introduce added complexity in design.

Table 1 provides an overview of representative studies on anisotropic scaffolds and compares their reported mechanical properties with those obtained in this study. As the table shows, many existing works achieve anisotropy but depend on complex manufacturing routes or non-standard materials, whereas the present approach uses FDM to achieve comparable results in a more straightforward and cost-effective way.

Despite significant progress in the field, most existing approaches still depend either on sophisticated manufacturing methods such as Selective Laser Melting (SLM) or cryo-printing, or on computational optimization frameworks that are difficult to reproduce^15,16^. By contrast, the current study offers an experimentally validated and easily reproducible strategy based on stretch-modified octet-truss lattices, fabricated using low-cost FDM. Through systematic variation of stretch ratio and unit cell size, the aim is to identify scaffold configurations that replicate the anisotropic modulus and yield strength of mandibular trabecular bone, providing a practical basis for next-generation maxillofacial implants.

**Table 1:** Comparison of mechanical properties of anisotropic scaffold designs.

Reference	Scaffold type/Method	Material	Young’s Modulus (GPa)	Yield Strength (MPa)	Anisotropy ratio (E_normal/E_stretched)
Liu et al.^8^	Machine Learning-based inverse design	Polymer (simulated)	~ 0.6–1	~ 15–25	~ 2–3
Cheikho et al.^9^	Graded porosity scaffold	Hydrogel/ Polymer	~ 0.3–0.6	~ 10–18	~ 1.5–2
Zhu et al.^10^	Cryo-printed anisotropic scaffold	Hydrogel	~ 0.2–0.5	~ 8–12	~ 2
Wang et al.^11^	Hierarchical lattice	Polymer composite	~ 0.7–0.9	~ 20–30	~ 2–2.5
Wei et al.^12^	Biomimetic scaffold	Hydrogel/ Polymer	~ 0.25–0.4	~ 7–12	~ 1.5–2
Present study	Stretched octet-truss (FDM)	Poly Lactic Acid	~ 0.44–0.8	~ 12–28	1.3–2.75

## Methodology

### Scaffold design and geometry

**Fig. 1:**
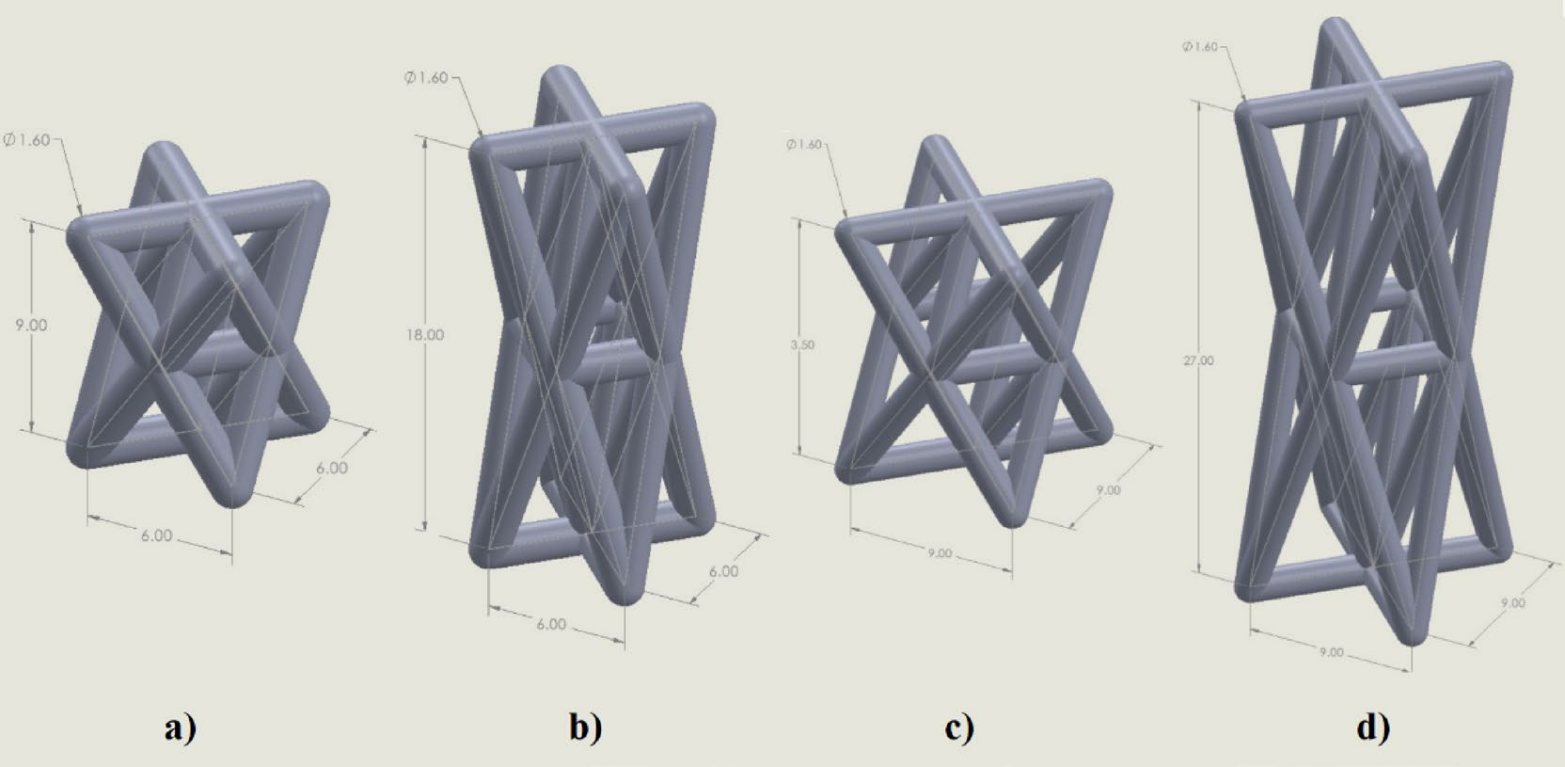
Four anisotropic unit cell models. (**a**) 6 × 6 mm in XY plane and 1.5× in the Z-direction; (**b**) 6 × 6 mm in XY plane and 3× in the Z-direction; (**c**) 9 × 9 mm in XY plane and 1.5× in the Z-direction; (**d**) 9 × 9 mm in XY plane and 1.5× in the Z-direction.

In this study, we adopted a stretch-modified octet-truss unit cell to design anisotropic bone scaffolds. The conventional octet-truss lattice, known for its stretch-dominated behavior, was geometrically stretched in the vertical direction to induce anisotropy. Two stretch ratios were investigated: 1.5 and 3 in the Z-direction. For each ratio, two sizes of unit cell base (6 mm × 6 mm and 9 mm × 9 mm) were selected, resulting in four distinct scaffold designs. The sizes of unit cell base, 6 × 6 mm and 9 × 9 mm, were selected based on a combination of factors. These dimensions fall within the resolution limits of the FDM 3D printer, ensuring reliable fabrication without significant print defects. At the same time, they correspond to pore sizes that are mechanically feasible while being comparable to the reported range of trabecular spacing in mandibular bone^17^. This dual consideration allowed us to balance manufacturability with biological and mechanical relevance. Figure 1 shows these four models. All struts had a circular cross-section of 1.6 mm diameter, chosen to match the 0.4 mm nozzle of the FDM printer and ensure fully solid rods.

To enable fair mechanical comparison across samples, each scaffold was designed to have a consistent final volume by repeating unit cells as needed. The arrangement and repetition counts for each model were computed using a MATLAB script, ensuring equal height and footprint for compression testing.

### CAD modeling

**Fig. 2:**
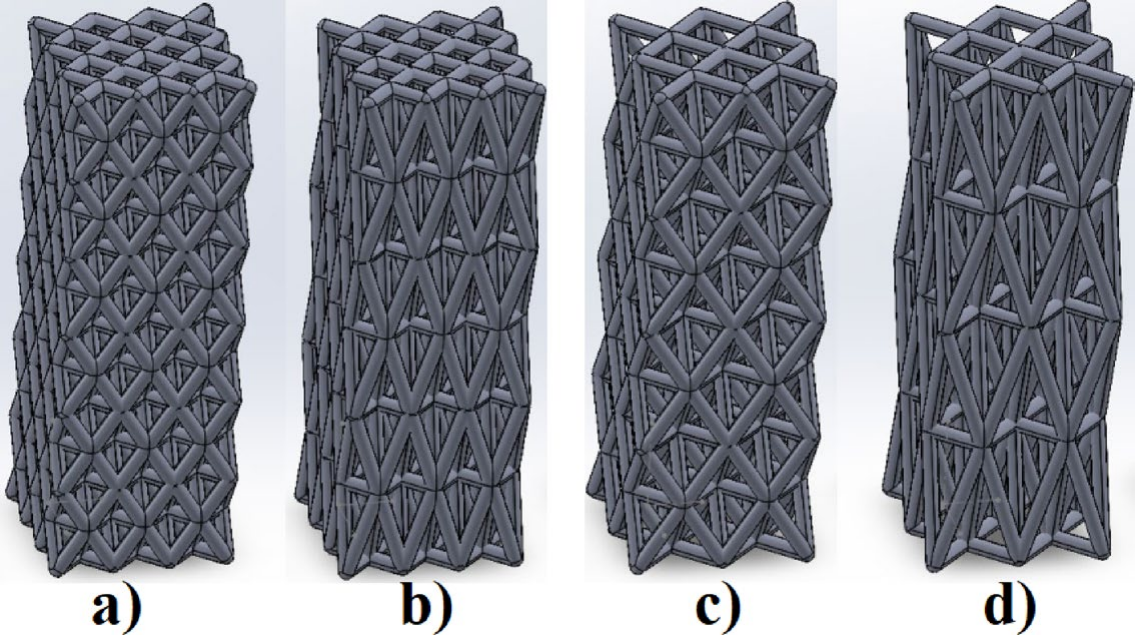
Four anisotropic full scaffold models. (**a**) Model 1; (**b**) Model 2; (**c**) Model 3; (**d**) Model 4.

The scaffolds were modeled using SolidWorks 2024 (Dassault Systems SolidWorks Corp., Waltham, MA, USA) Computer Aided Design (CAD) software. First, a conventional octet-truss unit cell was constructed by creating cylindrical struts at the vertices and edges of a cubic geometry using the “3D Sketch” and “Sweep” tools. The anisotropy was introduced by scaling the unit cell along the Z-axis using the “Scale” feature, with stretch ratios of 1.5 and 3 applied. The resulting unit cells were then replicated in the X, Y, and Z directions using the “Linear Pattern” command to form full scaffolds with dimensions of 18 × 18 × 54 mm. Finally, the solid models were exported in STL format for slicing and fabrication. Each unit cell was constructed as a central regular octahedron surrounded by eight regular tetrahedral, forming a spatially periodic lattice. The stretched versions were generated by scaling the unit cell in the Z-direction before repeating them to form the full scaffold. Four scaffold models were created and labeled Model 1 to Model 4 based on stretch ratio and base size. Models’ properties are shown in Table 2. Figure 2 shows full scaffolds by repeating the unit cells.

**Table 2:** Properties of unit cell models.

Model	Unit Cell Size (mm)	Stretch Ratio	Strut Diameter (mm)
1	6 × 6 × 9	1.5	1.6
2	6 × 6 × 18	3	1.6
3	9 × 9 × 13.5	1.5	1.6
4	9 × 9 × 27	3	1.6

### Printing of specimens

**Fig. 3:**
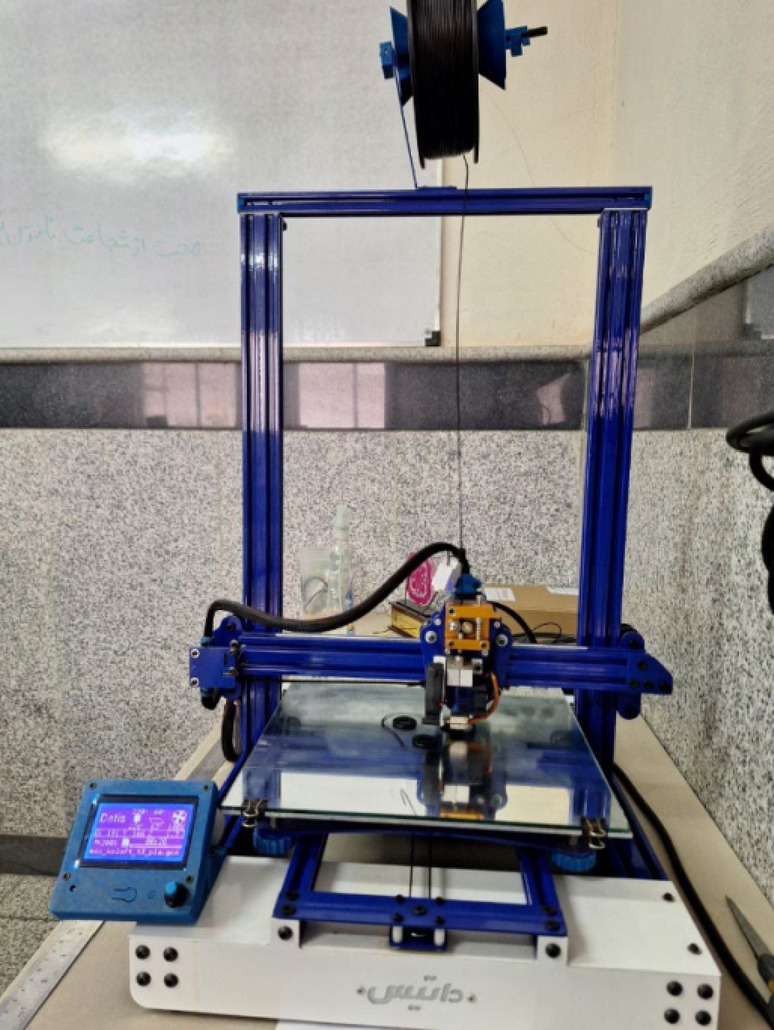
Datis Pro FDM 3D printer (Datis Co., Tehran, Iran) used for scaffold fabrication.

All models were fabricated using a Datis Pro FDM 3D printer (Datis Co., Tehran, Iran) equipped with a direct-drive extrusion system and a 0.4 mm nozzle. The printer is depicted in Fig. 3. The slicing process was performed in Ultimaker Cura 5.10.2 (Ultimaker B.V., Utrecht, Netherland) software with the following settings:

- Layer height: 0.3 mm.
- Wall thickness: 0.8 mm.
- Infill density: 0% (hollow struts).
- Nozzle temperature: 210 °C.
- Bed temperature: 90 °C.
- Print speed: 25 mm/s.
- Cooling fan: Enabled.
- Retraction: Enabled.

**Fig. 4:**
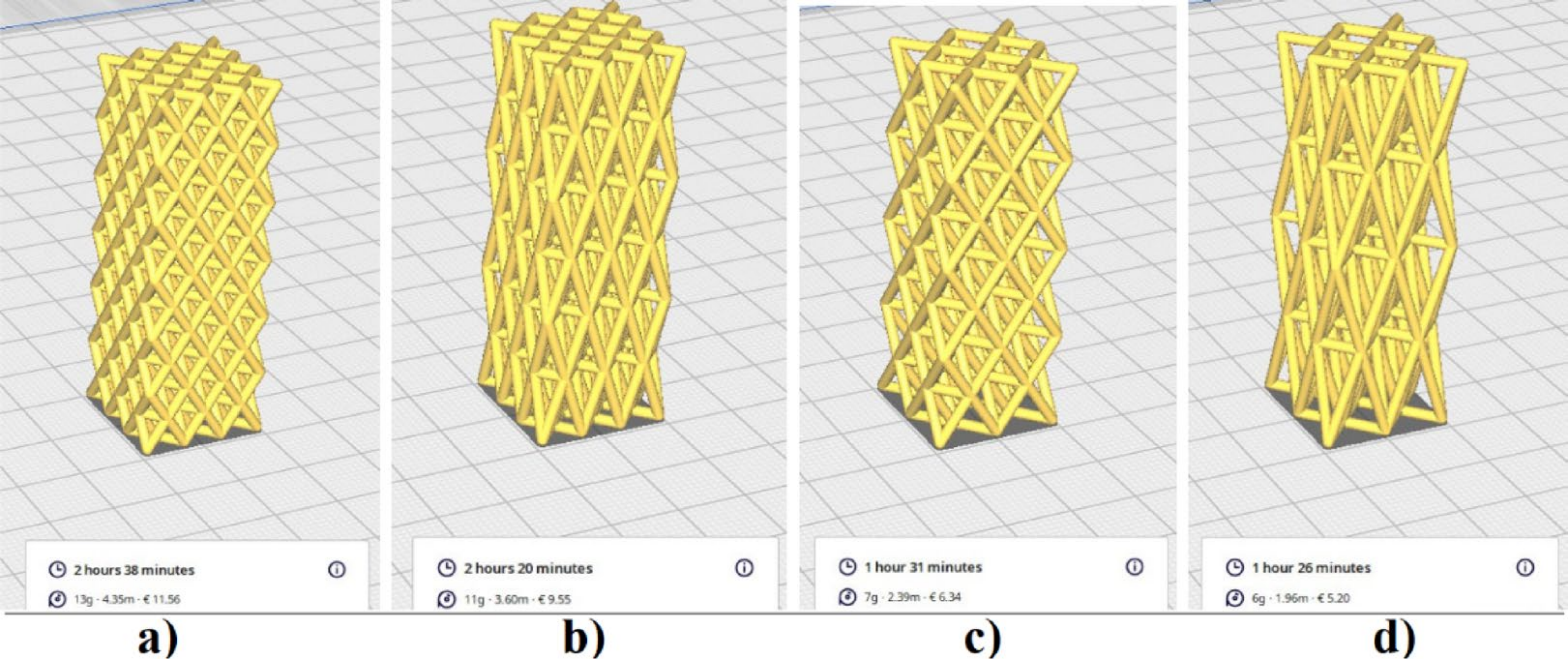
Arrangement and slicing of the four models in Ultimaker Cura 5.10.2 (Ultimaker B.V., Utrecht, Netherland). (**a**) Model 1; (**b**) Model 2; (**c**) Model 3; (**d**) Model 4.

The prints were made using eSUN’s PLA+ (Polylactic Acid Plus) (Shenzhen eSUN Industrial Co., Ltd., Shenzhen, China) filament. All models were oriented vertically during printing, with the stretching direction aligned along the Z-axis—the same axis later used for compression in the testing phase. The selected printing parameters (layer height, nozzle and bed temperature, print speed, and infill setting) were chosen based on previous studies on PLA scaffolds^18^. as well as the technical limitations of the Datis Pro FDM printer. These conditions ensured complete strut formation, dimensional accuracy, and reproducibility of the printed scaffolds. Figure 4 illustrates the setup in Ultimaker Cura, along with key specifications for the four selected models.

### Density measurement

The mass of each printed scaffold was measured using a precision scale, and its apparent density was calculated by dividing the mass by the external volume of the scaffold. Results are shown in Table 3.

**Table 3:** Specifications of each printed model.

Model	Mass (g)	Volume (mm³)	Apparent Density (kg/m³)
1	13.0	17,496	743
2	10.7	17,496	612
3	7.1	17,496	406
4	5.8	17,496	332

### Compression testing

Compression tests were conducted in accordance with the general principles of International Organization for Standardization (ISO 13314; Compression test for porous and cellular materials), adapted to the geometry and dimensions of the lattice scaffolds used in this study^19^. Similar quasi-static compression tests on polymer-based lattice structures have been reported in the literature, such as ABS-M30i scaffolds^20^, providing methodological consistency and validation. Deviations from the standard, such as specimen dimensions, internal lattice porosity, and strut-based architecture, are described below.

Unlike the solid cylindrical or cubic specimens typically specified in ISO 13,314, the present samples were architected lattice scaffolds with through-holes and non-standard external dimensions, designed in CAD to match the target scaffold geometry. While the shape and size deviated from the standard, the loading direction, strain rate, and methods for determining elastic modulus and yield strength were consistent with the ISO 13,314 procedure.

Engineering stress (σ) was calculated as the applied load (F) divided by the initial external cross-sectional area ($\:{A}_{0}$​) of the specimen, while engineering strain (ε) was obtained as the displacement change (Δh) divided by the initial specimen height ($\:{h}_{0}$​). These definitions follow the methodology outlined in ISO 13,314 for porous and cellular materials:

1 $$\:\sigma\:=\:\frac{F}{{A}_{0}}$$

2 $$\:\varepsilon\:=\:\frac{\varDelta\:h}{{h}_{0}}$$

**Fig. 5:**
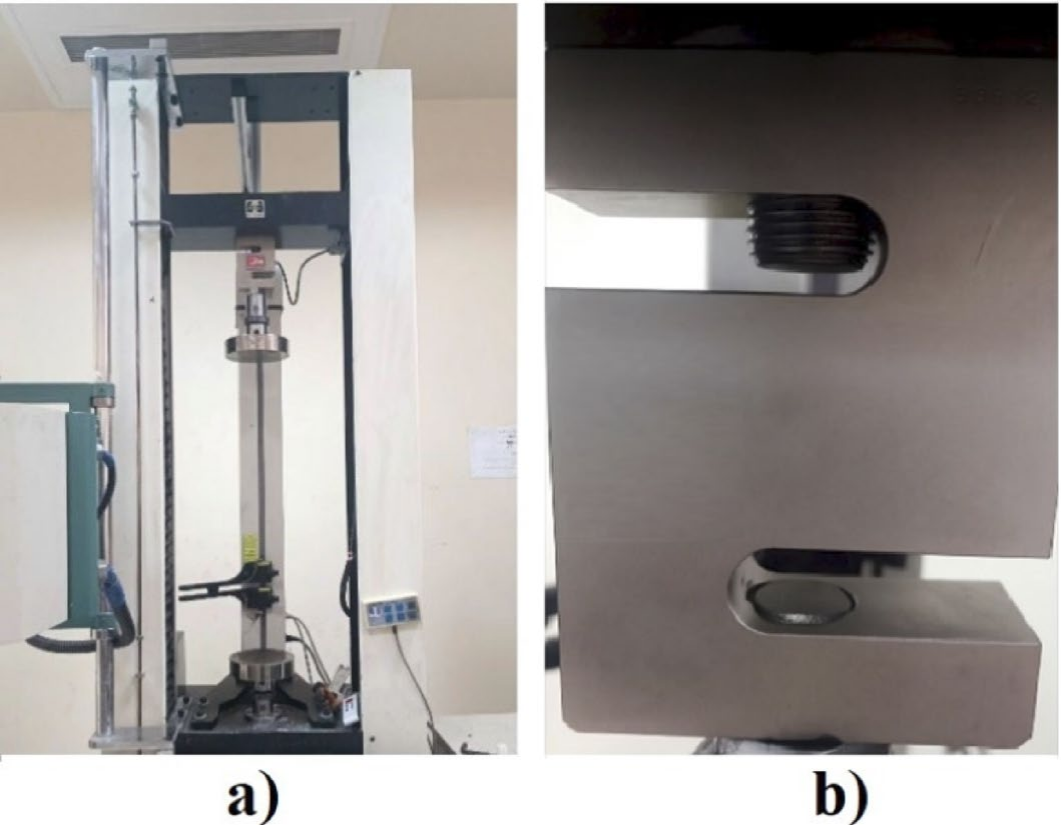
Compression test equipment. (**a**) SANTAM STM-50 universal testing machine (SANTAM Co., Tehran, Iran); (**b**) 5 ton Bongshin load cell (Bongshin Co., Seoul, South Korea).

Quasi-static uniaxial compression tests were carried out using a SANTAM STM-50 universal testing machine (SANTAM Co., Tehran, Iran) equipped with a 5 ton-Bongshin load cell (Bongshin, Seoul, South Korea). Figure 5 shows the used test machine. Prior to loading, a small preload of 5–10 N was applied to ensure full contact between the specimen and platens. The platens were aligned to minimize eccentric loading, following ISO 13,314 recommendations. The tests were performed at a constant crosshead speed of 1 mm/min, following standard compression protocols. The crosshead speed was set to 1 mm/min, corresponding to a nominal strain rate of ~ 0.005 s⁻¹ based on the initial specimen height^21,22^. This value was selected to minimize viscoelastic effects in PLA and to match ISO 13,314 guidelines for quasi-static loading^23,24^. Each scaffold was tested in two orientations: (1) loading applied along the stretching direction (Z axis), and (2) loading applied perpendicular to the stretching direction (X or Y axis). For each scaffold model and loading direction, compression tests were conducted on *n* = 3 specimens, and the average values of modulus and strength across replicates were reported.

Force-displacement data were recorded throughout the test and converted to engineering stress and strain by dividing the force by the initial cross-sectional area and displacement by the undeformed height of the scaffold. Young’s modulus was extracted from the linear region using linear curve fitting, and yield stress was defined using the 0.2% offset method. Representative stress–strain curves were recorded during compression tests, and key parameters such as modulus and yield strength were derived from these curves. Raw data were smoothed using the moving average method in MATLAB before analysis.

## Results and discussion

### Mechanical behavior under Quasi-Static compression

To evaluate the mechanical behavior of the designed scaffolds, quasi-static compression tests were conducted using the SANTAM STM-50 testing machine with a 5-ton Bongshin load cell at a crosshead speed of 1 mm/min. Each of the four scaffold designs was tested in two directions: parallel and perpendicular to the stretching axis. This allowed a direct comparison of their anisotropic properties.

**Fig. 6:**
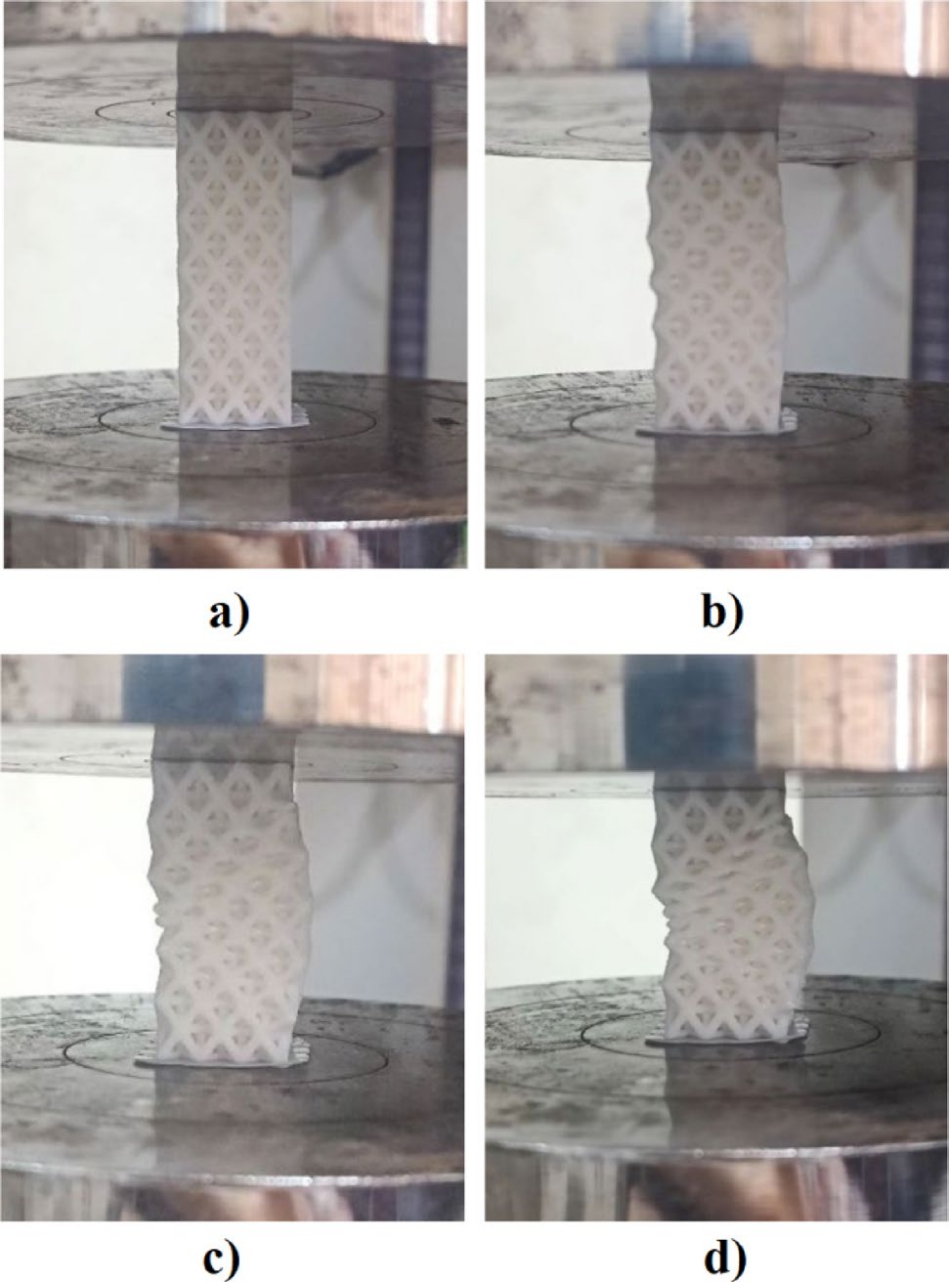
Four deformation stages during compression tests. (**a**) Elastic region; (**b**) Nonlinear transition; (**c**) Damage initiation; (**d**) Densification.

**Fig. 7:**
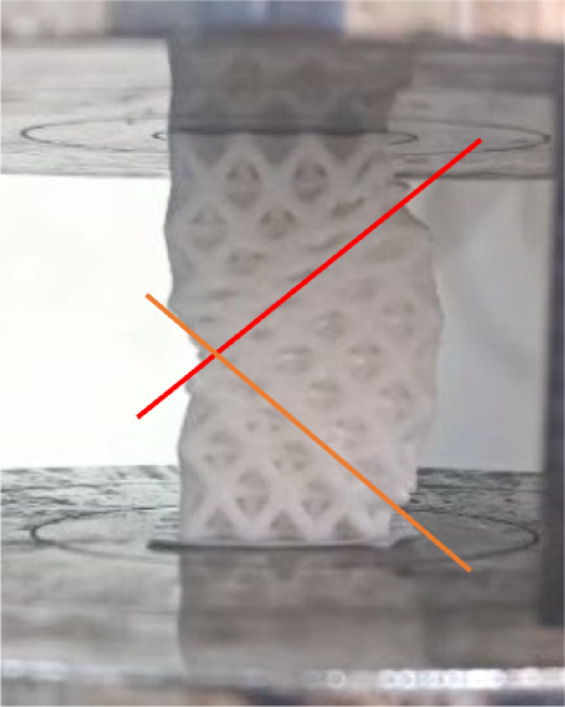
Representative images showing shear band formation at ~ 45° angles during compression tests of the scaffolds.

Figure 6 shows the engineering stress-strain behavior for the stretched octet-truss samples. All samples exhibited four characteristic deformation stages: elastic region, nonlinear transition, damage initiation, and densification^25,26^. A sudden drop in stress was observed after the elastic-plastic transition, corresponding to shear band formation at ~ 45° angles^27,28^, as shown in Fig. 7. This was consistent with maximum shear stress planes observed in cellular solids under compression^29^. The deformation stages observed here are consistent with prior studies on architected lattices^30^, including investigations under high strain rates which demonstrated comparable mechanical responses and energy absorption characteristics^31^.

### Elastic modulus and yield stress

The elastic modulus was extracted from the linear region of the smoothed force–displacement data using a moving average filter and polynomial curve fitting in MATLAB, while the 0.2% offset method was applied to determine yield strength^32^:

3 $$\:{\sigma\:}_{{y}_{0.2}}=E\:(\varepsilon\:-0.002)$$

**Fig. 8:**
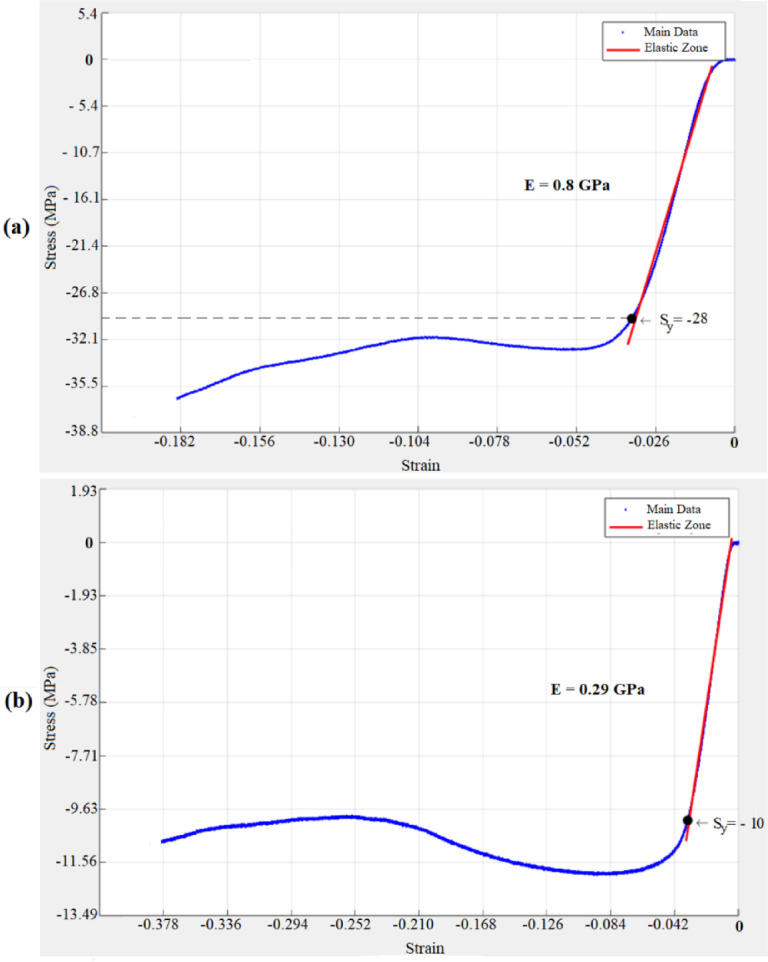
Representative stress–strain curves obtained from compression testing of the octet-truss scaffolds. (**a**) Model 1 loaded in the normal direction; (**b**) Model 1 loaded in the stretched direction.

In this equation, $\:{\sigma\:}_{{y}_{0.2}}$, $\:E$, and $\:\varepsilon\:$ represent yield strength, elastic modulus, and strain, respectively. The yield strength was determined using the 0.2% offset method, in which a line parallel to the initial linear portion of the stress–strain curve (slope = $\:E$) is drawn starting at a strain of 0.002. The intersection of this line with the experimental stress–strain curve defines the yield strength, in accordance with ISO 13,314 for porous and cellular materials. The quantitative results for all four scaffold models, including both normal and stretched loading directions and their corresponding apparent densities, are presented in Table 4 for direct comparison^33^. For clarity, throughout this manuscript the “stretched direction” refers to loading parallel to the stretch axis (Z-axis), whereas the “normal direction” refers to loading perpendicular to the stretch axis (X- and Y-axes). The representative stress–strain curves are presented in Fig. 8, illustrating the characteristic regions of elastic response, nonlinear transition, and densification. The mechanical properties summarized in Table 4 were obtained from these curves.

**Table 4:** The quantitative results for all four scaffold models.

Model	Unit Cell Size (mm)	Stretch Ratio	Direction	Elastic Modulus (GPa)	Yield Stress (MPa)	Apparent Density (kg/m³)
1	6 × 6	1.5	Normal	~ 0.80	~ 28	743
			Stretched	~ 0.29	~ 10	
2	6 × 6	3.0	Normal	~ 0.63	~ 21	612
			Stretched	~ 0.47	~ 16	
3	9 × 9	1.5	Normal	~ 0.52	~ 18	406
			Stretched	~ 0.19	~ 7	
4	9 × 9	3.0	Normal	~ 0.44	~ 15	332
			Stretched	~ 0.31	~ 12	

**Fig. 9:**
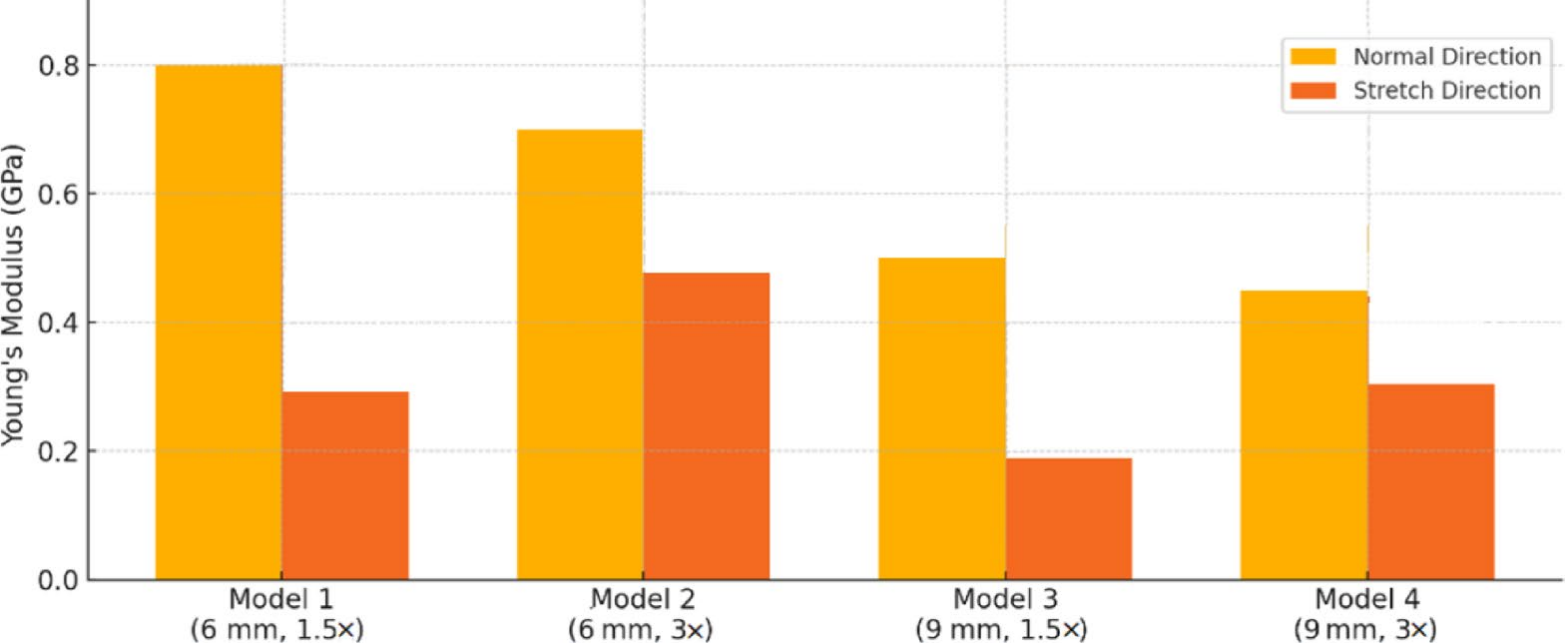
Comparison of Young’s modulus in the two directions.

As can be seen from Table 4, the stretched truss structures exhibited a clear anisotropic mechanical response. For Models 1 and 3 (stretch ratio = 1.5), the elastic modulus in the normal direction was approximately 2.75 and 2.53 times higher, respectively, than in the stretched direction. In contrast, for models with a 3 stretch ratio (Models 2 and 4), the modulus ratios were significantly lower (1.34 and 1.42), indicating diminished anisotropy. This trend was consistent with previous reports showing that unit-cell stretch ratio strongly affects directional stiffness and anisotropy in lattice scaffolds^34^. This trend is visually illustrated in Fig. 9, where the superiority of moderately stretched designs (Models 1 and 3) in achieving directional stiffness is evident.

**Fig. 10:**
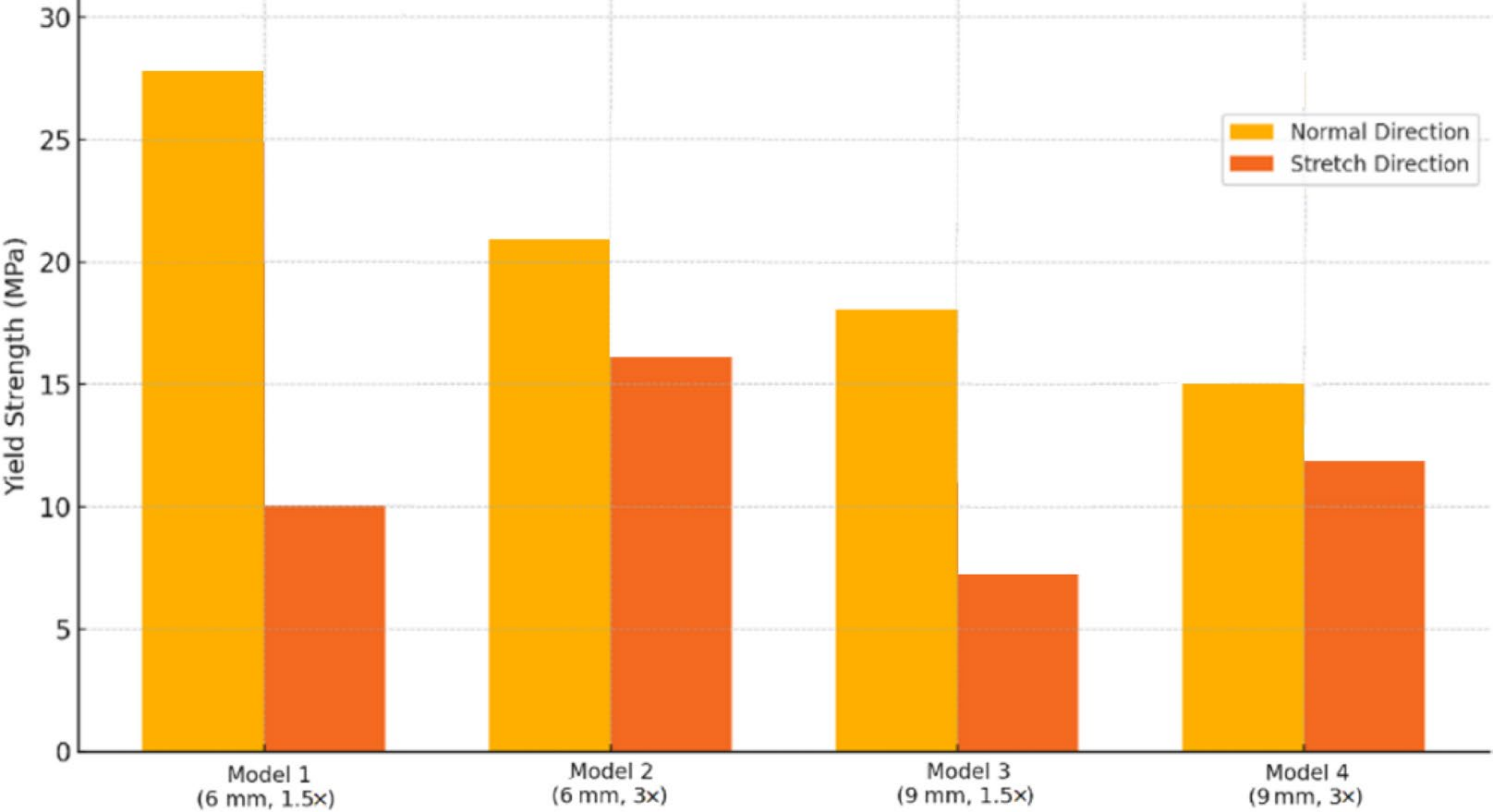
Comparison of yield strength in the two directions.

The yield strength results followed a similar pattern. Models 1 and 3 exhibited yield stresses in the normal direction approximately 2.8 and 2.57 times greater, respectively, than in the stretched direction, whereas Models 2 and 4 showed smaller directional differences. These findings are depicted in Fig. 10, further highlighting the consistent advantage of moderate stretching in reproducing the anisotropic mechanical behavior of mandibular trabecular bone. This observation was consistent with previous studies reporting that moderate unit-cell stretching improves anisotropic mechanical matching with trabecular bone^35^.

### Effect of unit cell size and stretch ratio

**Fig. 11:**
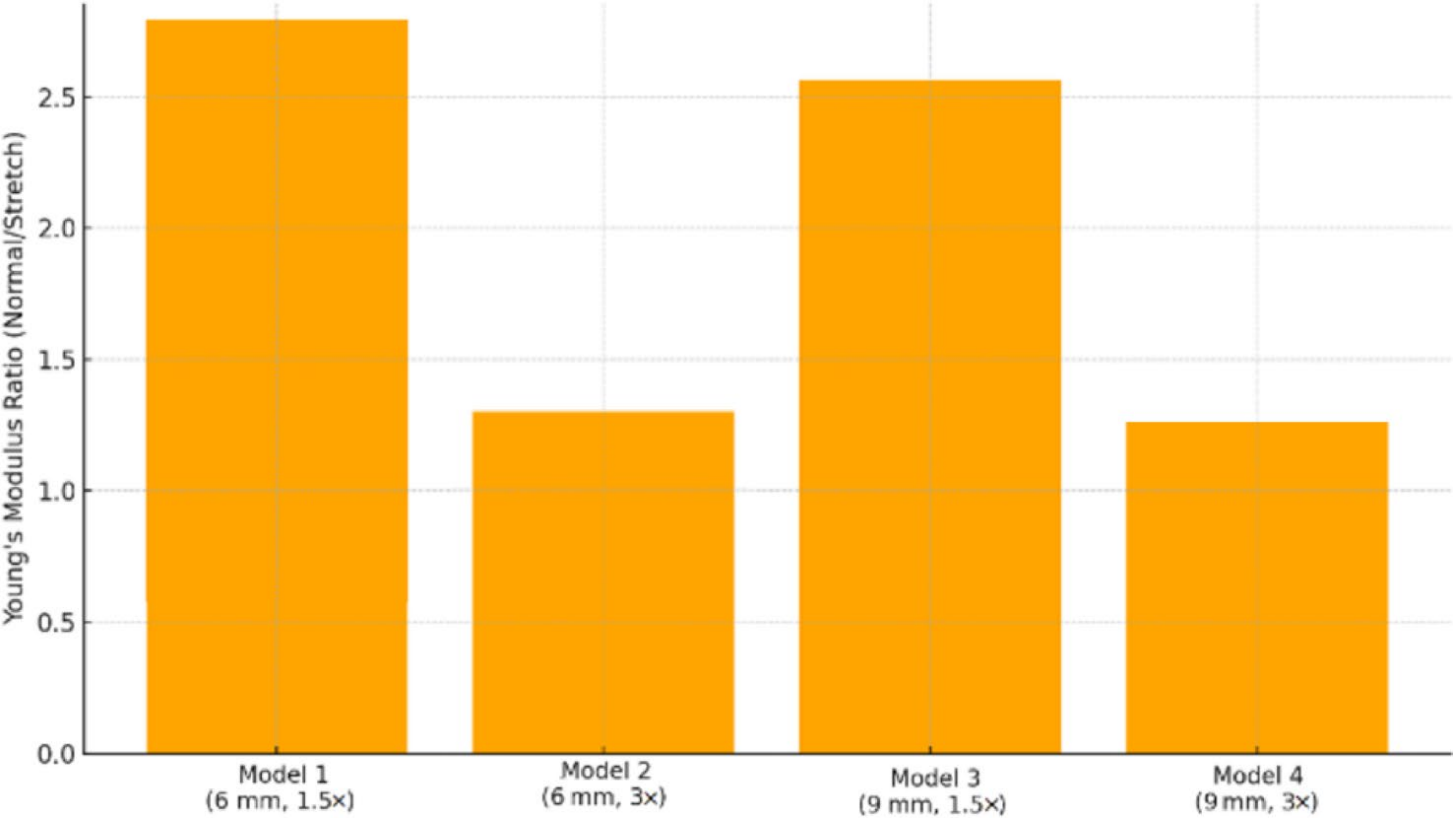
Anisotropy ratio of Young’s modulus.

Larger unit cells (9 mm) exhibited lower stiffness and yield strength due to the increased slenderness of the struts, which led to more pronounced bending and buckling. Moreover, the anisotropy ratio (E_normal/E_stretched) was most distinct in the moderately stretched (1.5×) designs. The calculated anisotropy ratios for the four scaffold designs are presented in Fig. 11. The highest ratio was observed for Model 1 (2.75), followed closely by Model 3 (2.53), confirming the pronounced directional stiffness in moderately stretched designs.

Model 1 demonstrated the highest mechanical performance and anisotropy ratio, making it the most suitable candidate for mimicking the direction-dependent properties of trabecular jawbone. Model 3 also showed a good balance between density and anisotropy, while model 2, despite a higher density than model 3, failed to exhibit significant directional stiffness variation.

### Comparison with jawbone properties

**Fig. 12:**
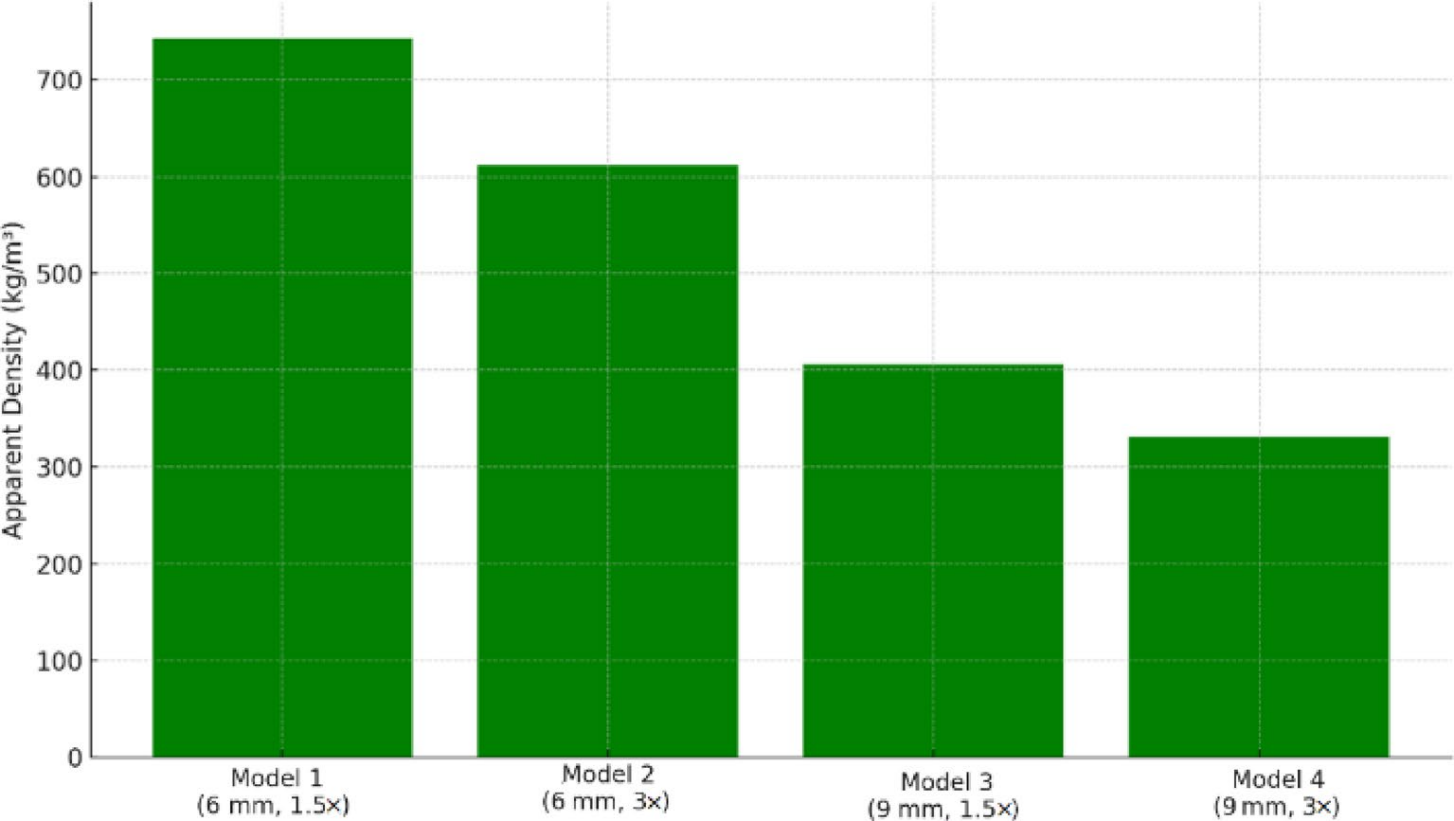
Apparent density of different scaffold models.

The anisotropic mechanical properties of the scaffolds were compared with those reported for mandibular trabecular bone. Literature values have suggested Young’s modulus of approximately 1.2 GPa in the longitudinal direction and 0.8 GPa in the transverse direction, with yield strengths ranging from 20 to 30 MPa^36,37^. Model 1 approached the lower bound of these values in the normal direction, with a modulus of ~ 0.8 GPa and a yield stress of ~ 28 MPa, indicating its potential suitability for dental implant scaffold applications. Its apparent density (743 kg/m³) was also within the range of natural jawbone (typically ~ 530–800 kg/m³ depending on location and age)^38^. The apparent densities of all four scaffold models are shown in Fig. 12, indicating that Models 1 and 2 fell near the upper bound of the jawbone density range, whereas Models 3 and 4 were closer to the lower bound.

**Fig. 13:**
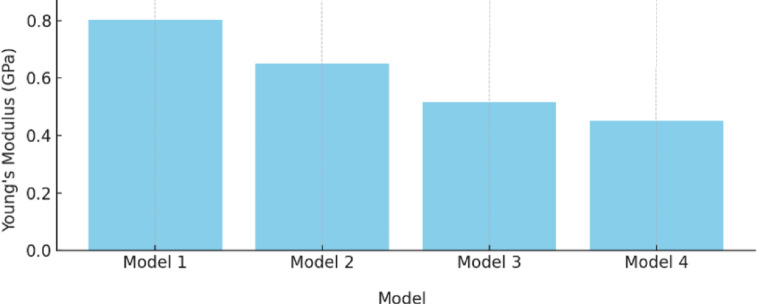
Young modulus vs. apparent density for different scaffold models.

When Young’s modulus was plotted against apparent density (Fig. 13), the results showed a trend consistent with the Gibson–Ashby scaling laws for cellular solids^39^. Models 1 and 3 occupied a favorable position within the target range for mandibular trabecular bone, balancing stiffness and density in a way that was likely to reduce stress shielding while maintaining mechanical compatibility. This relationship further supported the selection of moderately stretched designs as optimal candidates for maxillofacial scaffold applications.

#### Theoretical interpretation

The variations in stiffness and strength can be explained using the Gibson–Ashby model for cellular solids, which links normalized modulus and strength to the relative density of the structure^40^:

4 $$\:\frac{{\sigma\:}^{*}}{{\sigma\:}_{s}}=C\left(\frac{{\rho\:}^{*}}{{\rho\:}_{s}}\right)$$

5 $$\:\frac{{E}^{*}}{{E}_{s}}=C\left(\frac{{\rho\:}^{*}}{{\rho\:}_{s}}\right)$$

In this model, $\:{E}_{s}$ ​, $\:{\sigma\:}_{s}$, and $\:{\rho\:}_{s}$ represent the static modulus of the cellular structure’s bulk material, yield stress of the cellular structure’s bulk material, and density of the cellular structure’s bulk material (PLA in this case), respectively, Also $\:{E}^{*}$, $\:{\sigma\:}^{*}$, and $\:{\rho\:}^{*}$ represent The static modulus of the cellular structure, The yield strength of the cellular structure, and density of the cellular structure, respectively, and $\:C$ represents Gibson-Ashby constant, the value of which is dependent on the unit cell geometry. As illustrated in Fig. 13, the experimental data followed the expected power-law scaling, indicating that the mechanical performance of the scaffolds was primarily dictated by their relative density and internal cell geometry.

The pronounced anisotropy in Models 1 and 3 can be attributed to the stretch-dominated behavior of the octet-truss architecture in the normal direction and the increased slenderness of struts in the stretched direction, leading to bending-dominated deformation under perpendicular loading^41^.

#### Limitations

This study has certain limitations that should be acknowledged. First, the scaffolds were fabricated from PLA, which, while biocompatible, has lower stiffness and strength than permanent implant materials such as titanium alloys or bioresorbable composites reinforced with hydroxyapatite; results may therefore vary with alternative materials^42,43^. Second, the study was limited to quasi-static compression tests; fatigue and cyclic loading—both essential for evaluating long-term implant performance—were not investigated^44^. Third, all experiments were carried out under standard laboratory conditions rather than in a physiological setting (37 °C, high humidity), which could affect the mechanical response of polymers^45^. Finally, although the printed lattice geometries were designed to mimic mandibular trabecular architecture, they were scaled for experimental practicality and may require further refinement before direct patient-specific applications can be realized^46^.

## Immunological perspective

The success of maxillofacial scaffolds depends not only on mechanical mimicry of jawbone anisotropy but also on their immunological performance after implantation. Macrophages dominate the early host response, and a well-regulated shift from the pro-inflammatory M1 phenotype to the pro-regenerative M2 phenotype is essential for osseointegration rather than fibrotic encapsulation^47^.

Recent studies have highlighted that both scaffold composition and structure can modulate immune outcomes. For instance, a mussel-inspired polydopamine-coated gold–hydroxyapatite nanocomposite (PDA@Au-HA) was shown to inhibit M1 polarization, promote M2 phenotype, scavenge reactive oxygen species, and enhance osteogenesis via macrophage–preosteoblast interactions^48^.

Temporal immune strategies also show promise. A sequential delivery scaffold releasing lipopolysaccharide for early M1 activation followed by strontium ions for M2 switching effectively steered macrophage behavior and improved bone regeneration in vivo^49^. Surface-mediated immunomodulation is another powerful tool. For example, calcium phosphate coatings on melt-electrowritten scaffolds dynamically influenced macrophage polarization toward regenerative phenotypes^50^. Additionally, metabolic cues within scaffold design can direct immune responses. A citrate-functionalized scaffold suppressed glycolytic activity and favored M2-polarization through metabolic reprogramming of macrophages, thereby promoting bone homeostasis^51^.

In the context of material selection, it is important to consider that the present scaffolds were fabricated from polylactic acid (PLA). PLA is a Food and Drug Administration (FDA)-approved, biodegradable polymer valued for its printability and biocompatibility. However, its degradation into lactic acid can activate immune cell metabolic reprogramming—boosting pro-inflammatory cytokine expression and potentially leading to fibrosis or delayed integration^52,53^. Additionally, hybrid composites of PLA with Hydroxyl Apatite (PLA/HA) have been shown to evoke variable immune responses depending on HA morphology and size^54^. Another study highlighted that PLA hydrolysis products may contribute to inflammatory osteolysis mediated by oxidative damage through macrophage activity^55^. These findings underscore the need to consider PLA’s immunological limitations and explore mitigation strategies—such as buffering coatings, PLA composites, or controlled degradation—to support regenerative outcomes^56^.

In this study, PLA scaffolds were fabricated and evaluated for anisotropic mechanical performance; however, no direct macrophage polarization or cytokine assays were performed. The immunological effects discussed here are derived from recent literature on PLA-based biomaterials. Future incorporation of approaches such as PLA/HA composites, bioactive coatings, or ionic functionalization could mitigate pro-inflammatory responses and improve the translational potential of PLA scaffolds similar to those investigated in this work^52,56^.

Beyond material effects, scaffold geometry and anisotropy also influence immune cell behavior. Recent experiments reveal that macrophages respond differently to oriented architectures: elongated strut/pores promote macrophage elongation and spreading, which is associated with M2-like polarization, whereas isotropic or randomly oriented scaffolds tend to maintain more rounded, pro-inflammatory phenotypes^57^. Moreover, a scaffold with multiscale anisotropic architecture demonstrated enhanced anti-inflammatory macrophage polarization and bone regeneration in vivo, linking anisotropy to immune modulation^58^. These findings support the notion that geometry-driven cues can modulate macrophage phenotype independent of material composition.

To quantitatively link mechanical anisotropy and immune modulation, we introduce the anisotropy ratio (AR) definition:

6 $$\:AR=\frac{{E}_{normal}}{{E}_{\text{s}\text{t}\text{r}\text{e}\text{t}\text{c}\text{h}\text{e}\text{d}}}$$

In this equation, $\:{E}_{normal}$ ​and $\:{E}_{stretched}$ represent the elastic modulus in normal and stretched directions, respectively. Structures with higher AR (e.g. AR > 2.0) create directional mechanical environments that favor cell alignment and elongation along the stiff axis, which in turn promotes M2 polarization via mechanotransductive cues. This geometry–immunity coupling complements material-based immunomodulation.

These findings also suggest potential applications in patient-specific scaffold design. By integrating medical imaging (e.g., CT-based reconstruction) with computational tools such as finite element (FE) optimization, the anisotropic lattice parameters could be tailored to individual patient anatomy and loading conditions, further improving clinical outcomes.

## Conclusion

In this work, architected scaffolds inspired by stretched octet-truss geometries were designed, additively manufactured, and mechanically characterized to emulate the anisotropic behavior of mandibular trabecular bone. By systematically varying unit cell dimensions (6 mm and 9 mm) and stretch ratios (1.5 and 3), the directional stiffness and strength of the scaffolds were effectively tailored.

Experimental results demonstrated that the 1.5 stretch ratio in both 6 mm and 9 mm unit cells produced pronounced anisotropy, with elastic modulus ratios of 2.75 and 2.53, and yield strength ratios of approximately 2.8 and 2.57, respectively. These values deviate less than 7–10% from reported anisotropy in human mandibular bone, while the corresponding apparent densities of 743 kg/m³ (Model 1) and 406 kg/m³ (Model 3) lie within the physiological range of 530–800 kg/m³. Such close correspondence in mechanical and density parameters indicates strong potential for mitigating stress shielding and enhancing implant integration.

Compared with isotropic scaffolds, the proposed anisotropic designs offer directional load-bearing capability aligned with physiological demands. The results confirm that moderate directional stretching of octet-truss unit cells can generate bioinspired scaffolds with tunable anisotropic properties, thereby meeting critical requirements for maxillofacial and dental implant applications. Importantly, by mimicking the anisotropic mechanical behavior of mandibular trabecular bone, the designed scaffolds help to reduce stress shielding. This biomechanical compatibility facilitates more natural load transfer between the implant and host tissue, thereby enhancing clinical outcomes and long-term stability.

Future investigations should address the incorporation of composite materials such as PLA + HA or other bioresorbable polymers to enhance mechanical performance and bioactivity, evaluation under simulated physiological conditions through in-vitro testing to assess fatigue resistance and osseointegration, integration of patient-specific geometries via CT/MRI-based modeling combined with finite element optimization, and exploration of hybrid manufacturing strategies to produce clinically relevant scaffold sizes with localized reinforcement.

From an immunological perspective, while PLA provides a reproducible and cost-effective platform, its degradation products may promote early pro-inflammatory macrophage activity, potentially delaying integration. Incorporating hydroxyapatite reinforcement, bioactive ion release, or metabolic modulators has been shown to shift macrophage polarization toward regenerative M2 phenotypes, improve angiogenesis, and accelerate osteointegration.

Therefore, future PLA-based anisotropic scaffolds should evolve into immune-instructive composites capable of achieving both mechanical fidelity and controlled immune integration, offering a promising pathway toward next-generation, patient-specific constructs for mandibular bone regeneration.

## Data Availability

All research data have been embedded in manuscript main context.
